# Time-lapse observation of mouse preimplantation embryos using a simple closed glass capillary method

**DOI:** 10.1038/s41598-023-47017-8

**Published:** 2023-11-14

**Authors:** Yasuyuki Kikuchi, Daiyu Ito, Sayaka Wakayama, Masatoshi Ooga, Teruhiko Wakayama

**Affiliations:** 1 Faculty of Life and Environmental Science, University of Yamanashi, Kofu, 400-8510 Japan; 2 Advanced Biotechnology Center, University of Yamanashi, Kofu, 400-8510 Japan; 3 Department of Animal Science and Biotechnology, School of Veterinary Medicine, Azabu University, Fuchinobe, Chuo-ku, Sagamihara, 252-5201 Japan

**Keywords:** Embryology, Time-lapse imaging

## Abstract

Time-lapse observation is a popular method for analyzing mammalian preimplantation embryos, but it often requires expensive equipment and skilled techniques. We previously developed a simply and costly embryo-culture system in a sealed tube that does not require a CO_2_ incubator. In the present study, we developed a new time-lapse observation system using our previous culture method and a glass capillary. Zygotes were placed in a glass capillary and sunk in oil for observation under a stereomicroscope. Warming the capillary using a thermoplate enabled most of the zygotes to develop into blastocysts and produce healthy offspring. This time-lapse observation system captured images every 30 min for up to 5 days, which confirmed that the developmental speed and quality of the embryos were not affected, even with fluorescence. Overall, this new system is a simple time-lapse observation method for preimplantation embryos that does not require dedicated machines and advanced techniques.

## Introduction

Preimplantation embryo analysis is important for studying the mechanisms of embryonic developmental biology. In early studies, embryos were collected from living animals at each developmental stage because they could not be cultured in vitro. However, in 1934, mammalian embryos were successfully cultured in vitro^1^, and Whitten and Biggers later developed a simple chemically defined medium to enable the development of one-cell embryos up to the blastocyst stage in vitro in mice^2^. This led to significant progress in mammalian embryology. However, mammalian embryos require high CO_2_ concentrations during development^3^ and must be protected from light exposure^4^, similar to somatic cells such as fibroblasts^5,6^; therefore, they are typically cultured in CO_2_ incubators without light. To examine embryo development, embryos are taken from the incubator and observed under a microscope in air, which may stress the embryos and reduce their quality, resulting in lower developmental rates to blastocysts^7^. Therefore, time-lapse observation or live-cell imaging technology has been developed to allow observation of embryos without compromising their quality^8,9^.

In 1929, time-lapse observation of mammalian embryos was first successful in rabbits^10^, but the observation period was short and in vivo collected embryos were used. Since then, time-lapse observation has been improved to allow an extended culture period with less damage to the cells and embryos^11^ and is now used for many other species^12–15^. Time-lapse observation is commonly used for analyzing the developmental speed and morphology of embryos in human assisted reproductive technology and allows for prediction of embryo fate at the preimplantation stage, which can aid in selecting the most viable embryos for transfer^16,17^.

Time-lapse observation typically requires skilled technique to overcome several factors such as light intensity, shooting time intervals, and decision making about the embryos. Although recent advancements have automated and simplified these adjustments, the required equipment remains expensive. In addition, observation from the 1-cell to the blastocyst stage takes about 1 week, which means that the equipment is occupied for an extended period for one experiment. During this time, not only do researchers need to pay attention to the room temperature and vibration, but it is also difficult to conduct experiments that compare several different culture conditions because the experiment can only be performed once a week. Consequently, only large clinics and research institutions with substantial funding can perform detailed embryo analysis and selection using live-cell imaging equipment. Hence, many laboratories, including those in developing countries, are unable to conduct these experiments.

We previously developed the “Optimized Medium with CO_2_ Pressure and Sealed-tube culture method”, which is abbreviated to the “Ops culture method”^18^. This is a simple method for culturing embryos in a sealed container, such as a PCR tube, without a CO_2_ incubator. It has been used successfully used to transport live embryos to other laboratories using a water bottle^18^ and was even used on the International Space Station to determine the effect of microgravity on embryo development^19^. Building on this success, we sought to establish a simple and efficient time-lapse observation system using the Ops culture method and a time-lapse microscope camera. Because observing embryos through the wall of a PCR tube is challenging, we used a glass capillary instead, which has high transparency but no gas permeability. Additionally, the capillary can be easily sealed with a burner at both ends. Previous studies have shown that glass capillaries do not have a toxic effect on the development of mammalian embryos cultured without a CO_2_ incubator^20,21^.

In this study, we aimed to develop a simple and efficient time-lapse observation method for mammalian embryos using the Ops culture method with sealed glass capillaries and a thermoplate on a microscope. First, we determined how to seal the embryos into the capillary to observe their development. Next, we cultured mouse zygotes using several commercial glass capillaries in a thermostat chamber and selected the optimal capillary. Finally, we performed time-lapse observation of the zygotes developing into blastocysts in the glass capillary, comparing their developmental rate, cleavage speed, and offspring rate to those obtained using a dedicated equipment. Additionally, we established fluorescence imaging for short-term observations. Our study demonstrated that time-lapse observation of embryos using a glass capillary is a simple and efficient analysis method.

## Results

### Establishing the method for observing embryos in a glass capillary

**Figure 1:**
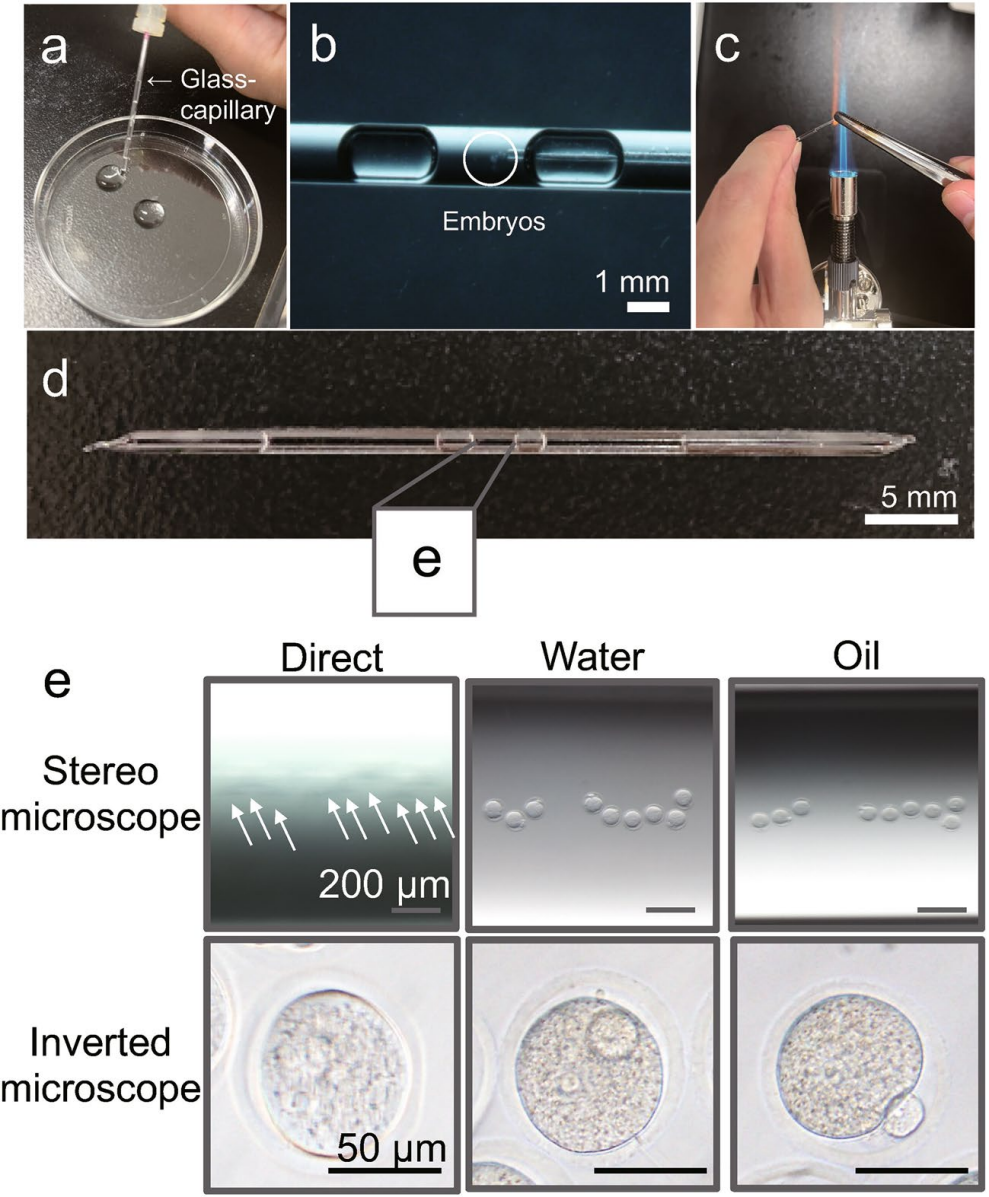
Observation of embryos using a glass capillary. To enclose embryos in a glass capillary, OptC medium was added to the glass capillary using a syringe, and two air layers were added (**a**). Embryos were then inserted between the air layers using a mouth pipette (**b**). Next, both ends of the capillary were sealed using a burner (**c**). The resulting glass capillary with embryos is shown in (**d**). To observe embryos, we used both a stereomicroscope and inverted microscope (**e**). However, when we attempted to observe the embryos using direct placement (left), the images were blurred. To address this issue, we submerged the glass capillary in a dish of water (center) or oil (right). Scale bar: 1 mm (**b**), 5 mm (**d**), 200 µm (Stereo microscope), 50 µm (Inverted microscope).

First, we aimed to determine the most suitable method for sealing embryos in glass capillaries for time-lapse observation. We prefilled a glass capillary with Optimized CO_2_ containing (OptC) medium (Fig. 1a) and inserted one-cell stage zygotes obtained from in vitro fertilization (IVF) into the capillary using a mouth pipette from the side (Fig. 1b). However, when we tried to seal the ends of the capillary with a burner (Fig. 1c), the area near the edges became too hot for the embryos. In addition, it was challenging to regulate the positions of embryos, often they get together in the same place. To address these issues, we introduced air bubbles beside the embryos inside the glass capillary (Fig. 1d), a technique which we used for subsequent experiments. We observed that the embryos appeared blurry when viewed through the glass capillary using stereomicroscopy or inverted microscopy, especially with the former. To overcome this, we placed the glass capillary in a dish of water or oil, which provided better clarity when viewing the embryos (Fig. 1e).

### Selecting a glass capillary suitable for embryo culture

**Figure 2:**
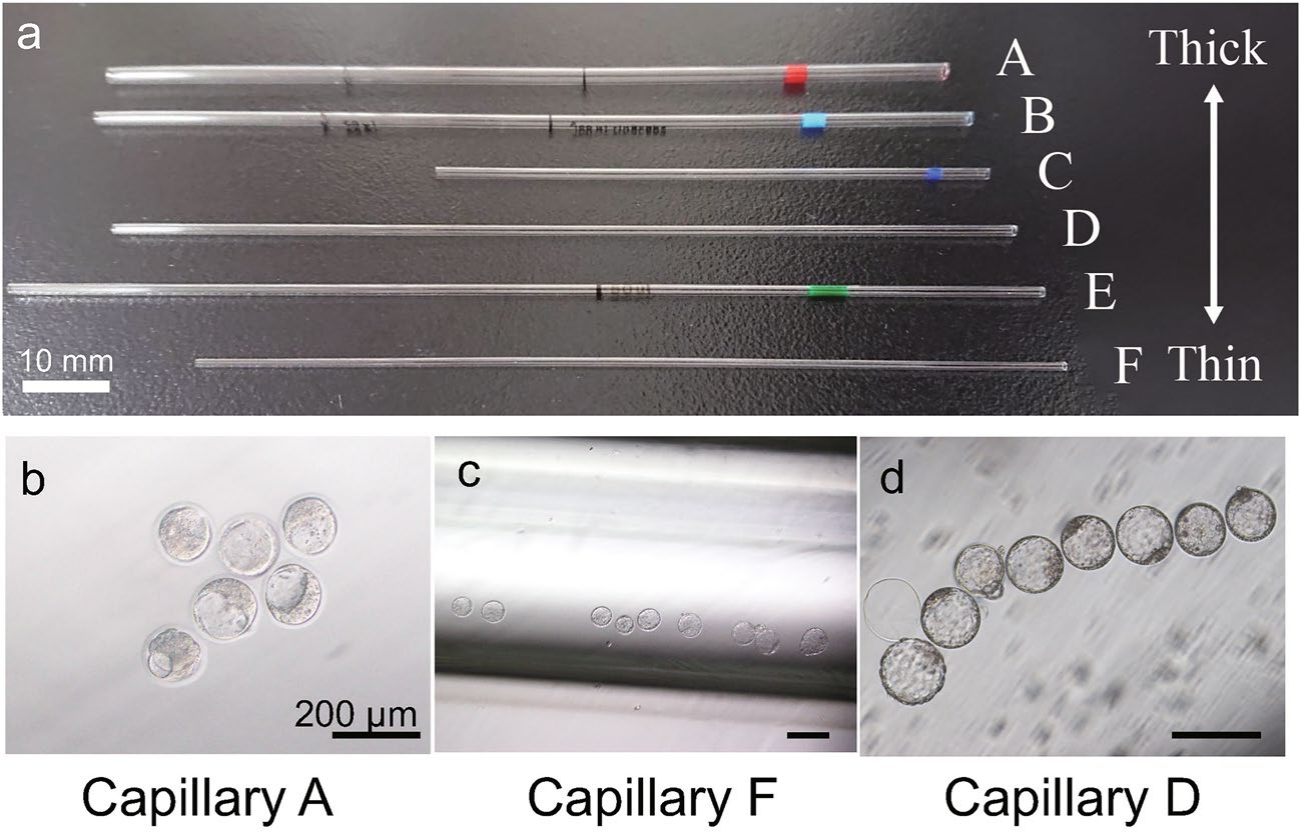
Determining a suitable glass capillary for embryo culture and observation. (**a**) To determine the optimal glass capillary for cultivation and observation, we compared six commercial glass capillaries. Scale bar: 10 mm (**b–d**) Blastocyst development in the indicated glass capillaries after 96 h of culture. Picture resolution varied among capillaries. Scale bar: 200 µm.

We tested six commercially available glass capillaries with different inner diameters (Fig. 2a) to determine the most suitable capillary for embryo culture and observation. We compared the developmental rate and visibility of embryos derived from IVF in the capillaries to those cultured on a dish in a CO_2_ incubator (control group) after five days of culture in a thermostat chamber. As shown in Table 1, we found no significant difference in developmental rate to the blastocyst stage between the capillaries (different types and inner diameters) and the control (83.1–97.6% vs. 92.7%, p > 0.05). However, we observed differences in embryo handling and visibility depending on capillary type. Thick capillaries made it difficult to focus on the embryos (Fig. 2b) and seal the capillary end with a burner, whereas thin capillaries caused the embryos to align in a long row (Fig. 2c), requiring lower magnification to capture all embryos. Based on these findings, we chose glass capillary D (with a medium inner diameter), which had a high developmental rate and allowed for observation of several embryos simultaneously (Fig. 2d), and used it for subsequent experiments.

**Table 1:** Developmental rate of embryos cultured in glass capillaries with different diameters in a thermostat chamber.

Inner diameter of glass capillary (mm)	No. of embryos enclosed	No. of embryos observed	No. of embryos developed to various stages (%)			
			2-Cell	4–8-Cell	Morula	Blastocyst
Control	41	41	41 (100)	41 (100)	40 (97.6)	38 (92.7)
A: 1.89	45	41	41 (100)	40 (97.6)	40 (97.6)	40 (97.6)
B: 1.39	59	59	51 (86.4)	49 (83.1)	49 (83.1)	49 (83.1)
C: 1.2	39	39	39 (100)	39 (100)	38 (97.4)	34 (87.2)
D: 1.05	86	82	81 (98.8)	81 (98.8)	80 (97.6)	79 (96.3)
E: 0.93	30	30	30 (100)	30 (100)	30 (100)	27 (90)
F: 0.75	48	46	45 (97.8)	40 (87)	40 (87)	40 (87)

No significant differences were found between each group and the control (χ^2^ test: p > 0.05).

### Confirming that the thermoplate provides sufficient warmth for normal embryo development

**Figure 3:**
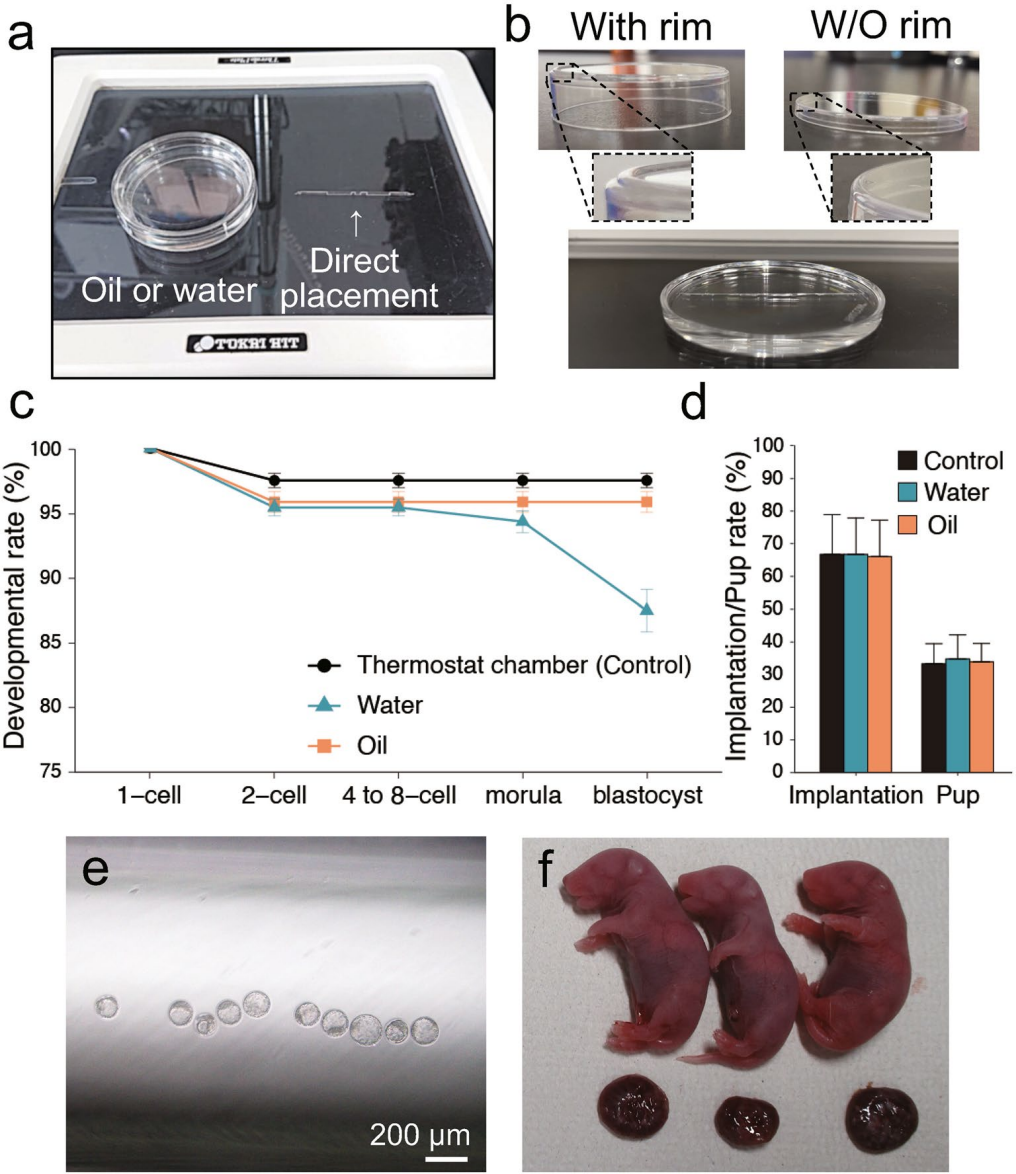
Developmental potential of embryos cultured on a thermoplate in glass capillaries. (**a**) Glass capillaries were warmed using a thermoplate. (**b**) First, a dish with a rim was used to warm the glass capillary (left). However, this dish did not efficiently transfer the temperature to the inside of dish. Thus, a dish without rim was used instead (right and bottom). (**c**) Developmental rate of embryos in glass capillaries cultured on a thermoplate. (**d**) Implantation and pup rates. (**c,d**) Black: thermostat chamber; blue: submerged in water; orange: submerged in oil; error bars: standard error of the mean. Blastocysts (**e**) and pups (**f**) derived from embryos cultured in glass capillaries using the developed method. Scale bar: 200 µm.

Next, to culture embryos with observation in glass capillaries, they were warmed using a thermoplate on a microscope. They were cultured for 96 h using three different warming methods: direct placement, immersion in water, and immersion in oil (Fig. 3a). Embryos derived from IVF warmed in glass capillaries by direct placement on the thermoplate had a similar high developmental rate as those cultured in the thermostatic chamber, whereas embryos in glass capillaries placed in water or oil exhibited significantly lower developmental rates (Table S1). Moreover, a stable developmental rate could not be achieved (ranging between 0 and 100%), likely due to warming by the thermoplate being affected by changes in room temperature despite trials of the heat retention methods, such as heated between two thermoplates (Table S2). We addressed this issue in two ways. First, we used a different type of dish that had a flat bottom without a rim, which facilitated more effective transmission of temperature to the water/oil in the dish (Fig. 3b). Second, we conducted the experiment in a small room to minimize fluctuations in room temperature caused by people entering and leaving frequently.

After changing the culture method, we obtained a higher and more stable developmental rate to the blastocyst and pup stages (Fig. 3c–f, Tables S3, S4), which was comparable with the control group. Notably, when we attempted to use this method in a temperature-controlled room at 20 °C, almost all examined embryos developed to the blastocyst stage (Table S3). Based on these results, we used this culture system for time-lapse observation.

### Performing time-lapse observation using the new method

**Figure 4:**
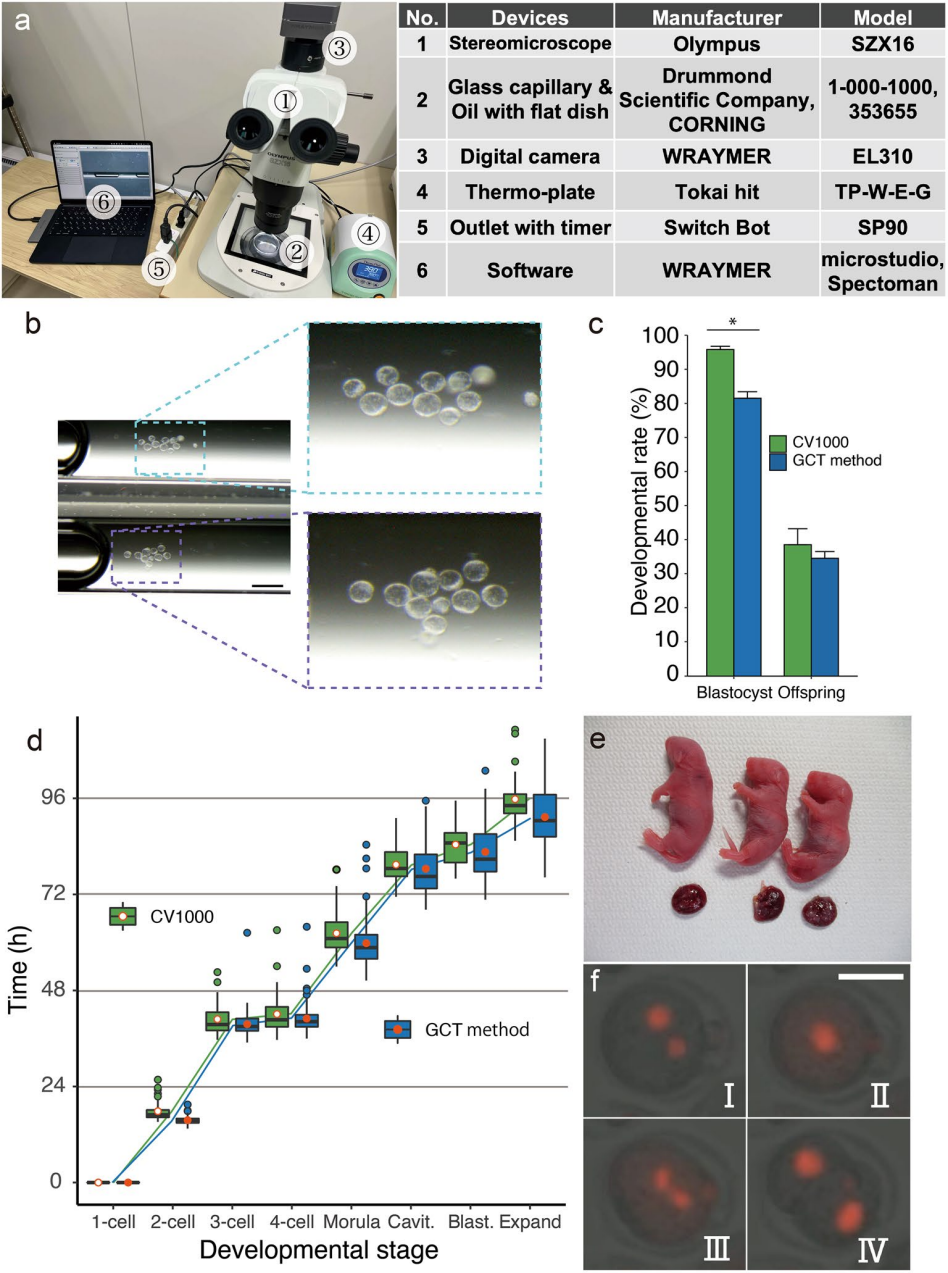
Comparison of embryo quality between the capillary time-lapse observation system and conventional equipment. (**a**) Photographs (left panels) and list (right table) of the equipment used for new glass capillary-based time-lase observation method. (**b**) Images shot using the GCT method at 96 h after IVF. This method could shoot two capillaries in the same photo without reducing imaging quality. Scale bar: 500 µm. (**c**) Developmental rate of embryos to the blastocyst or offspring stage. Green and blue bars indicate embryos cultured using the CV1000 and GCT method, respectively. *Indicate significance difference was found (p < 0.05); error bars: standard error of the mean. (**d**) Developmental speed of embryos in the CV1000 and GCT methods. Red dot: mean; Each group color dots: outliers; *Cavit.* Cavitation, *Blast.* blastocyst. (**e**) Pups obtained using the GCT method after time-lapse observation for 5 days. (**f**) Photographs shot using the fluorescence-based GCT method. Zygote was shot at the 2PN stage (**I**), nuclear fusion (**II**), nuclear fission (**III**), whereas the embryo was shot at the two-cell stage (**IV**). Red fluorescence indicates histone H2B-mCherry. Scale bar: 50 µm.

Finally, we attempted to perform time-lapse observation using the new simplified method. We used a stereomicroscope, simple digital camera for general use, thermoplate, and Switch Bot Plug, which controlled the emission of light (Fig. 4a). Such equipment might already be available in an embryo research laboratory but could otherwise be acquired with limited expense. By combining these devices, a simple time-lapse observation system was created to capture time-lapse images of embryos in culture under a microscope, with the light turned on only while the images are being taken.

The captured images can be saved in the cloud using individual or shared computers, enabling the creation and editing of movies if necessary. We termed this new simple system the “Glass Capillary Time-lapse observation” method (GCT method). Using this method, we performed time-lapse observation of zygotes developing into blastocysts, capturing images every 30 min up to 5 days (Fig. 4b). During shooting, the microscope stage was darkened using a blackout. We used the timer plug to turn on the light for only 1 min during each shoot to analyze the developmental rates and speeds of embryos. We then collected blastocysts from glass capillaries and transferred them into recipient females to detect their potential for full-term development. We compared the results obtained from the GCT method with those obtained using a dedicated live-cell imaging machine (CV1000).

Using the GCT method, 81.5% of zygotes developed into blastocysts, which was slightly but significantly lower than the control (81.5% vs. 95.8%, p < 0.01; Table 2, Fig. 4c). However, the results obtained via the GCT method were clear enough to evaluate cleavage and observe morphology even if two glass capillaries were observed simultaneously under the same stereomicroscope (Fig. 4b). When blastocysts were hatching, it was sometimes difficult to track embryos using the CV1000, but the GCT method was able to capture many embryos within the angle of view (Movies S1, S2).

**Table 2:** Time-lapse observation of embryo development in vitro.

	No. of embryos enclosed	No. of embryos developed to various stages (%)			
		2-Cell	4–8-Cell	Morula	Blastocyst
CV1000	143	143 (100)	143 (100)	142 (99.3)	137 (95.8)
GCT method	168	168 (100)	164 (97.6)	152 (90.5)	137 (81.5)*

*Indicates a significant difference (χ^2^ test: p < 0.05) compared with the blastocyst development rate using the CV1000-based method.

We compared developmental speed until the blastocyst stage using the photos obtained from each system. The GCT method was slightly quicker, taking 15 h and 34 min to reach the two-cell stage from IVF, whereas the CV1000 took 17 h and 49 min (Fig. 4d, Table S5). Using the GCT method, embryos took 82 h and 37 m to reach the blastocyst stage, which is comparable to the time taken when embryos were cultured using the CV1000 (84 h and 26 m) (Fig. 4d). Moreover, the GCT method was able to obtain healthy pups at a rate similar to that of the control (34.5% vs 38.5%; Table 3, Fig. 4c,e).

**Table 3:** Full-term development of embryos to offspring observed using time-lapse observation methods.

	No. of embryos transferred (recipient)ª	No. of implantations (%)	No. of offspring (%)	Mean body weight (g)	Mean placenta weight (g)
CV1000	65 (7)	46 (70.8)	25 (38.5)	1.70 ± 0.32	0.15 ± 0.03
GCT method	58 (7)	49 (84.5)	20 (34.5)	1.83 ± 0.23	0.16 ± 0.05

ªSome of the blastocysts detailed in Table 2 were used as embryos.

No significant differences were found between the CV1000 (control) and GCT method in terms of implantation, offspring (χ^2^ tests: p > 0.05), body and placenta weights (t-test: p > 0.05).

Dispersions of body and placenta weight were evaluated by s.e.m.

We also attempted fluorescence live-cell imaging using the GCT method. We injected histone H2B-mCherry mRNA into oocyte, and then we performed intracytoplasmic sperm injection (ICSI) to produce a zygote, cultured them for 24 h before taking both bright field and fluorescent photographs using a general inverted fluorescent microscopy. The embryos cultured using this method successfully formed pronuclei and developed to the two-cell stage without affecting the developmental speed, despite being exposed to UV light (Table 4). With this observation, we were able to accurately identify the position of the pronucleus through fluorescence imaging (Fig. 4f, Movie S3).

**Table 4:** Fluorescence-based live-cell imaging of embryo development in vitro.

	No. of oocytes enclosed	No. of embryos stained	No. of embryos developed to different stages (%)		Average time to develop to different stages (hh:mm)	
			2PN	2-Cell	Nuclear fusion	2-Cell
CV1000	46	45	38 (82.6)	31 (81.6)	17:05	17:56
GCT method	49	46	42 (91.3)	37 (88.1)	17:20	18:52

No significant differences were found between the CV1000 (control) and GCT method in terms of the blastocyst development rate (χ^2^ tests: p > 0.05) and developmental time (t-test: p > 0.05).

## Discussion

We successfully developed a simple and efficiency time-lapse observation method, the GCT method, which has comparable developmental rates and speeds relative to a conventional time-lapse or live-cell imaging machine. The GCT method utilizes tools that already exist in institutions researching mammalian embryos, making it an accessible option for laboratories with limited research funding. Additionally, the GCT method can be used with a standard computer, and images can be saved to cloud storage, allowing remote work and simplification of experiments.

The maintenance of room temperature is critical for the GCT method. Embryos cultured without air conditioning often stopped developing at the two-cell stage (Table S2). When room temperature is unstable, a temperature sensor with the thermoplate controls heating, resulting in rapid and frequent changes in the temperature of the glass capillary and embryos, which the embryos may not withstand. Stress during the one-cell stage seriously also affects zygotes^18^, sometimes lethally, even if the temperature change is temporary or for a short period. For example, when embryos are cultured in a 38 °C water bottle, the zygote can only be cultured for a maximum of two days because the temperature of the water bottle gradually drops. Still, if a thermostatic chamber is used, embryos can be developed into blastocysts. This study shows that embryo development is possible when embryos are cultured under stable temperature conditions, even if placed in glass capillaries and cultured on a microscope.

The conventional time-lapse observation or live-cell imaging method requires specific skills, such as aligning embryos regularly to reduce the number of shots, protecting embryos from contaminants, and setting focus, among other skills. When using conventional equipment to shoot time-lapse observation up to the hatching stage, it is necessary to make a hole in the dish or perform careful arrangement to avoid embryo escape. However, the GCT method is straightforward, with embryos placed in a sealed glass capillary, which prevents the entry of contaminants. Embryos are also naturally aligned and fall to the bottom of the capillary, making it easy to shoot them. By reducing the magnification, two glass capillaries can be observed by one stereomicroscope without interfering with the analysis (Fig. 4b), and the number of glass capillaries that can be observed at one time would be possible to increase by making the capillaries thinner. Notably, during this experiment, an earthquake of intensity three occurred (December 3, 2021, Kofu, Japan), but the GCT method was unaffected without a vibration removal board, and the embryo culture could continue, unlike the control group (conventional method) where almost all embryos escaped.

If time-lapse observations from zygote to blastocysts were made using conventional equipment (such as the CV1000), higher-resolution images could be obtained, but the experiment could only be done once a week. In contrast, our method has a lower resolution but can be easily implemented in any laboratory with a stereomicroscope. In addition, because the position of the embryos was unchanged in glass capillary, the time-lapse observation can be interrupted during observation and use the stereomicroscope for other purpose, then resumed the time-lapse observation of the embryos at the same position. Moreover, if a laboratory has several stereomicroscopes, each of them can be used for time-lapse observation simultaneously so that various data can be obtained at a low cost and in a short period. This method could be beneficial when making comparisons under multiple conditions, especially for preliminary experiments. For example, many checks of individual reagents that must be done prior to the preparation of culture media or differences among strains or age of mice. In particular, outbred mice, such as the ICR strain, which are easy to purchase and often used in laboratories with tight budgets, including our lab, have a difference in the speed of embryo development between individuals (Fig. 4d). In such cases, this method will be helpful because a large number of embryos must be measured for statistical analysis. Because the information obtained from time-lapse observation is an important assessment of embryonic development or quality, the ability to collect data efficiently and at a low cost is very attractive for basic embryology research.

Another limitation of GCT method is the fluorescence live-cell imaging component. It requires a lamp housing to stay running, and the exposure is controlled by a shutter, which markedly reduces the lifespan of the mercury lamp. Furthermore, the microscope filter cannot be changed in this method, limiting the ability to observe multiple fluorescence staining.

Our new method, the GCT method, will be easily adopted by institutions without prior experience in time-lapse observation or specialization in mammalian embryo analysis. Indeed, it is a simple and efficiency method that requires minimal setup and can be readily implemented without any complications. Moreover, we will work to improve the GCT method to make it more user-friendly and enhance its functionality.

## Materials and methods

### Animals

Eight-to-twelve-week-old ICR female and male, were obtained from the Shizuoka Laboratory Animal Center (Hamamatsu, Japan). The surrogate pseudo-pregnant ICR females that were used as recipients of the embryos were mated with vasectomised ICR males, whose sterility was previously demonstrated. On the day of the experiment or after having finished all experiments, mice were euthanised by cervical dislocation. All experiments were conducted according to the Guide for the Care and Use of Laboratory Animals and were approved by the Institutional Committee of Laboratory Animal Experimentation of University of Yamanashi with reference number: A4-10. All experiments were performed in accordance with these regulations and guidelines, which is followed in the ARRIVE guideline. All mice have been kept under SPF conditions, with controlled temperature (25 °C), relative humidity (50%), and photoperiod (14L-10D). They were fed a commercial diet and provided distilled water ad libitum. In this study, body weight was not measured except recipient female because body weight of young mice does not affect the quality of embryos.

### Preparing an optimized CO_2_ containing medium

We prepared an Optimized CO_2_ containing medium (OptC medium) before performing IVF or ICSI. Chatot-Ziomek-Bavister (CZB) medium^22^ dispensed into 5-mL plastic tubes were placed in a CO_2_ incubator (APN-30DR, ASTEC CO., Ltd., Fukuoka, Japan) with the lids loose for 24 h. Detailed preparation was described previously^18^.

### In vitro fertilization (IVF)

In vitro fertilization was performed as previously described^23^. Briefly, spermatozoa were collected from the cauda epididymitis of ICR, male mice into 200 μL of human tubal fluid (HTF) medium^24^ that was then covered with sterile mineral oil and capacitated by incubation for 1 h at 37 °C under 5% CO_2_ in a CO_2_ incubator. During sperm preincubation, cumulus–oocyte complexes (COCs) were collected from the oviducts of ICR female mice that were induced to superovulate by consecutive injections of 7.5 IU pregnant mare serum gonadotropin (Serotropin®, ASKA Animal Health Co., Ltd., Tokyo, Japan) and 7.5 IU human chorionic gonadotropin (hCG; 2413402X2053, ASKA Animal Health Co., Ltd.) 48 h apart. Sixteen hours after hCG injection, the mice were euthanised to collect COCs. After sperm preincubation, 5 μL aliquots of the suspension were added to droplets of HTF medium containing COCs. The final sperm concentration was approximately 1 × 10^5^ cells/mL. Fertilized zygotes, which were determined by the extrusion of second polar body and two pronuclei, were collected from the droplets and washed in CZB medium. The zygotes were temporarily placed in fresh droplets of CZB medium preincubated at 37 °C under 5% CO_2_ and cultured for subsequent experiments. All embryos used for the experiments except for fluorescence live-cell imaging were ones derived from IVF.

### Preparation of mRNA

After linearization of the template plasmids (pcDNA3.1-polyA83^11^) at the XhoI (histone H2B-mCherry), mRNA was synthesized using RiboMAX™ Large Scale RNA Production Systems-T7 (Promega, Madison). For efficient translation of the fusion proteins in embryos, the 5ʹ-end of each mRNA was capped using the Ribo m7G Cap Analog (Promega), according to the manufacturer’s protocol. To circumvent the integration of template DNA into the embryonic genome, the reaction mixtures for in vitro transcription were treated with RQ-1 RNase-free DNase I (Promega). Synthesized mRNA was treated with phenol–chloroform to remove protein components and then stored at − 80 °C until use.

### Microinjection of mRNA

When we performed fluorescence live-cell imaging, we prepared oocytes labelled histone fluorescent tag. Histone H2B-mCherry mRNA was diluted with nuclease-free water to 50 ng/μL before use. mRNA was injected into the cytoplasm of oocyte using borosilicate glass capillaries as described previously^25^. Briefly, microinjection was performed in HEPES-buffered CZB on an inverted microscope with a micromanipulator (Narishige, Tokyo, Japan). The zona pellucida and cytosolic membrane were penetrated with a piezo drive (PRIME Tech, Tokyo, Japan). Ten min after microinjection, the oocytes were washed and cultured in CZB. The mRNA-injected oocytes were incubated at 37 °C under 5% CO_2_ in air for at least 2 h after injection to allow time for protein production.

### Intracytoplasmic sperm injection (ICSI)

ICSI was performed as previously described^26^. Briefly, for the microinjection of spermatozoa, 1–2 µL of the sperm culture solution were directly moved to the injection chamber. The application of several piezo pulses was used to separate the spermatozoa head from the tail, and the head was then injected into the oocytes injected mRNA. The embryos that survived ICSI were incubated in the CZB medium at 37 °C with 5% CO_2_. Those embryos were used for fluorescence live-cell imaging.

### Embryo culture using a glass capillary

We prepared six kinds of commercially available glass capillaries, A; 9600222, AS ONE Corporation, B; 9600299, AS ONE Corporation, C; 05-760-0, Erma Inc, Saitama, Japan, D; 1-000-1000, Drummond Scientific Company, AP, US, E; 2-000-050, Drummond Scientific Company, F; BF150-75-10, SUTTER INSTRUMENT, CA USA. These glass capillaries information was described in Table S6. Glass capillaries were cut to about 45 mm because of fitting in a dish with rim (430589, CORNING, NY, US) or a flat bottom dish (353655, CORNING). OptC medium suck into glass capillary using a syringe with grip like a Fig. 1a. About 8 to 15 zygotes derived from IVF put into the center medium layer using a mouth pippet (Fig. 1b). After that, both ends of glass capillary were burned by gas burner and sealed medium and embryos (Fig. 1c). These embryos were warmed in 37 °C thermostat chamber (0040534-000, TAITEC CORPORATION, Saitama, Japan) or on 38 °C thermoplate (plate; TP-SZX2A or TP-W, Tokai Hit, Shizuoka, Japan, thermostat; TP-W-E-G, Tokai Hit) up to 96 h. In the case of thermoplate, we cultured embryos in glass capillary on directly placement or dish with water or oil to observe embryos. We performed this experience in 25 °C or 20 °C stable room. After culture, glass capillaries were wiped up by paper carefully. We wrapped capillaries paper why not to injured hands and cut both ends of capillaries by ampoule cutter. Embryos collected were moved to culture dish until embryo transfer.

### Preparation of time-lapse observation by GCT method

To light up of stereo microscope (SZX16, Olympus, Tokyo, Japan) only shooting time, we connected Switch Bot Plug (SP90, SwitchBot, Tokyo, Japan) to the outlet which enable to switch on or off by the time. We used microscope specialized camera (EL310, wraymer, Osaka, Japan) and this dedicated app (microstudio or Spectoman, wraymer) and shoot time-lapse photos according to the manufacture’s instructions and saved photos in Dropbox that why assume remote work. After that, glass capillary with embryos derived from IVF put into dish filled oil on the 38 °C thermoplate and we put black curtain over the microscope. We performed time-lapse observation form zygote to expand blastocyst by shooting every 30 min up to 4 or 5 days. We set turning on light only 1 min while every shooting by timer plug. As control, some of embryos were live-cell imaged using CellVoyager^TM^ CV1000 (Yokogawa, Tokyo, Japan). We transferred blastocysts cultured only 96 h into recipient’s female uterus.

### Fluorescence live-cell imaging by GCT method

We performed fluorescence observation by time lapse shooting using GCT method. We used inverted microscope (IX-71, Olympus) equipped Mercury Lamp housing (U-RFL-T, Olympus) and inverted microscope-specialized thermoplate (Tpi-110RH26, Tokai hit). We controlled light and laser by switchbot-bot (W0202200-GH, Switch Bot) by the time. Immediately after injection sperm to embryo overexpressed histone H2B-mCherry, these embryos were cultured by GCT method or CV1000 up to 24-h post insemination and shot pictures every 30 min. In the GCT method, bright field photos were white-balance adjusted after capture, then merged with fluorescent photos to create a movie. Thus, bright field and fluorescence photos were staggered for 5 min. We set turning on fluorescence laser only 3 s to decrease affection of laser irradiation to embryo.

### Embryo transfer

Embryo transfer was performed as previously described^27^. Blastocyst-stage embryos derived from embryos cultured in sealed plastic tubes were transferred into the uteri of pseudopregnant female ICR mice at 2.5 days post-coitum (dpc), which had previously been mated with vasectomized male ICR mice. On the day of embryo transfer, recipients were anesthetized by intraperitoneal injection of medetomidine, midazolam, and butorphanol. Between four and seven embryos were transferred into each uterine horn. Offspring were obtained at 18.5 dpc via cesarean section.

### Statistical analysis

The developmental speed and weight of pups and placenta were evaluated using a t-test, whereas the rates of embryo development, implantation, and the birth of offspring were evaluated using a χ^2^ test. p values of < 0.05 were considered statistically significant.

### Supplementary Information

Supplementary Movie S1.

Supplementary Movie S2.

Supplementary Movie S3.

Supplementary Information.

## Data Availability

All data generated or analysed during this study are included in this published article and are available from the corresponding author on reasonable request.
