# Supplementary material for: Time-lapse observation of mouse preimplantation embryos using a simple closed glass capillary method
Source: Sci Rep. 2023 Nov 14;13:19893. doi: 10.1038/s41598-023-47017-8 (PMC10646084; doi:10.1038/s41598-023-47017-8)
Supplement: Supplementary file 4 — Supplementary Information. [file 41598_2023_47017_MOESM4_ESM.pdf]

Time-lapse observation of mouse preimplantation embryos using a simple closed glass capillary method

Yasuyuki Kikuchi[1], Daiyu Ito[1], Sayaka Wakayama[2], Masatoshi Ooga[1,3], Teruhiko Wakayama[2*]

[1] Faculty of Life and Environmental Science, University of Yamanashi, Kofu, 400-8510, Japan

[2] Advanced Biotechnology Center, University of Yamanashi, Kofu, 400-8510, Japan

[3] Department of Animal Science and Biotechnology, School of Veterinary Medicine, Azabu University, Fuchinobe, Chuo-ku, Sagamihara, 252-5201, Japan.

[*] Correspondence: T. Wakayama (twakayama@yamanashi.ac.jp)

Table S1 Effects of different heating methods of glass capillary on embryo development.

Heating method		Set Temp. (°C)	No. embryo observed	No. embryo developed to (%)				
				2-cell	4 to 8-cell	Morula	Blast.	Min-Max
Thermostat chamber	Direct placement	37	57	57 (100)	55 (96.5)	55 (96.5)	54 (94.7)[a]	(78-100)
Thermoplate	Direct placement	37	63	63 (100)	63 (100)	63 (100)	49 (77.8)[b]	(29-100)
	Soaked in water	37	39	29 (74.4)	27 (69.2)	23 (59)	21 (53.8)[c]	(33-80)
	Soaked in oil	37	97	61 (62.9)	55 (56.7)	39 (40.2)	36 (37.1)[c]	(0-96)

[a, b, c] these characters indicated a significant difference (χ^2 test) compared in blastocyst rate ($P < 0.05$). Set Temp.; Set temperature. Blast.; Blastocyst.

Table S2 Culture in a glass capillary without air-conditioned room.

	Setting Temp. (ºC)	No. of zygote	2-cell	4-cell	M/B	Detail
Thermostat chamber or CV1000	37	9	9	9	9	
	37	9	9	9	9	
	37	8	8	8	8	
	37	10	10	10	10	
	37	8	8	8	8	
	37	10	10	10	10	
Thermoplate	37(upper)+37(lower)	18 (N=2)	18	0	0	Put thermoplate upper & lower sides
	37+37	9	9	0	0	Thermoplate upper & lower
	35.5+37.5	9	9	9	8	Thermoplate upper & lower
	37+37	9	9	0	0	Put thermoplate upper & lower sides
	37+37	8	8	8	8	Air-conditioned + thermoplate upper & lower
	37+37	30 (N=2)	28	2	0	Air-conditioned + thermoplate upper & lower

This culture system is week at change of room temperature especially in winter. So, we tried two solutions to maintain temperature. Firstly, we arranged air-conditioned experiment room. Secondly, we warmed dishes by putting upper and lower thermoplates.

Table S3 Developmental potential of embryos in glass capillary using flat dish and different room temperature.

Heating method		RT (ºC)	Set Temp. (ºC)	No. of observed embryo	No. embryo developed to (%)			
					2-cell	4 to 8-cell	Morula	Blast.
Thermostat chamber	Direct placement	-	37	79	77 (97.5)	77 (97.5)	77 (97.5)	77 (97.5)
Thermoplate flat dish	Soaked in water	25	38	87	83 (95.4)	83 (95.4)	82 (94.3)	76 (87.4)*
	Soaked in oil	25	38	72	69 (95.8)	69 (95.8)	69 (95.8)	69 (95.8)
	Soaked in oil	20	38	38	38 (100)	38 (100)	38 (100)	38 (100)

* asterisks indicated a significant difference (χ^2 test) compared in blastocyst rate with controls (P < 0.05).
RT; Room temperature, Set Temp.; Set temperature, Blast.; Blastocyst.

Table S4 Full-term development of embryo culture in glass capillary.

Heating method		No. embryo transferred [a] (recipient)	No. implantation (%)	No. offspring (%)	Mean body weight (g)	Mean placenta weight (g)
Thermostat chamber	Direct placement	45 (5)	30 (66.7)	15 (33.3)	1.81 ± 0.19	0.13 ± 0.02
Thermoplate	Soaked in water	69 (8)	46 (66.7)	24 (34.8)	1.83 ± 0.21	0.14 ± 0.03
	Soaked in oil (RT; 25ºC)	59 (6)	36 (66.1)	20 (33.9)	1.72 ± 0.28	0.15 ± 0.03

[a] Embryos were selected from parts of blastocysts derived from Table 3.
Dispersions of body and placenta weight were evaluated by s.e.m..
No significant difference each group to control in implantation and offspring (χ^2 test $p > 0.05$).
No significant difference each group to control in body and placenta weight (t-test $p > 0.05$).

Table S5 Developmental speed of embryo culture in glass capillary.

	Developmental time to each stage from *in vitro* fertilization (hh:mm)							
	1-cell	2-cell	3-cell	4-cell	Morula	Cavitation	Blastocyst	Expand
CV1000	0:00	17:49	40:49	42:05	62:18	79:26	84:26	95:47
GCT method	0:00	15:34	39:34	40:59	59:48	78:24	82:37	91:19

No significant difference GCT method to CV1000 in each developmental time (t-test $p > 0.05$).

Table S6 Kinds of glass capillary using culture.

	Inner diameter (mm)	Glass thickness (mm)	Medium volume (μL)	Corting
A	1.89	0.31	14.0	-
B	1.39	0.28	7.6	-
C	1.20	0.20	5.7	-
D	1.05	0.20	4.3	-
E	0.93	0.22	3.4	-
F	0.75	0.38	2.2	-
